# Navigating the online food environment: policy pathways for promoting food access, transparency, and healthy food choices online

**DOI:** 10.3389/fnut.2024.1473303

**Published:** 2024-10-08

**Authors:** Eva Greenthal, Katherine Marx, Emily Friedman, Sara John, Joelle Johnson, Christina LiPuma, DeAnna Nara, Sarah Sorscher, Karen Gardner, Aviva Musicus

**Affiliations:** Center for Science in the Public Interest, Washington, DC, United States

**Keywords:** food environment, food policy, food access, food labeling, food marketing, nutrition, online food retail, online food delivery

## Abstract

The internet is drastically changing how U.S. consumers shop for groceries, order food from restaurants, and interact with food marketing. There is an urgent need for new policies to help ensure that the internet is a force for good when it comes to food access, transparency, and nutrition. This article outlines actions that federal agencies—like the U.S. Department of Agriculture (USDA), U.S. Food and Drug Administration (FDA), and Federal Trade Commission (FTC)—and state and local governments can take to improve the online food environment. We recommend policies in three settings: online grocery retail, online restaurant ordering, and marketing on social media and other online platforms. For example, USDA could finalize regulations increasing access to online WIC and remove barriers to accessing online SNAP by requiring large retailers to waive online delivery and service fees for SNAP purchases. FDA could improve access to nutrition information by issuing guidance describing what product information should be available at the online point of selection. FTC could give better guidance on appropriate tactics when marketing to children and collect better data on how companies are marketing food to children online. Finally, state governments could pass laws like New York’s recently introduced Predatory Marketing Prevention Act to address false and misleading advertising of unhealthy foods aimed at children and other vulnerable groups.

## Introduction

The internet is drastically changing how U.S. consumers shop for groceries, order food from restaurants, and interact with food marketing. Nearly one in five U.S. consumers buys groceries online at least once per month (1). Two-thirds of restaurant sales are generated by orders placed online or by phone (2). Half of consumers (51%) say third-party apps are their preferred way to browse for food, and 86% order on third-party apps at least twice a month (3).

Online ordering and delivery present new opportunities to expand food access. For example, during the COVID-19 pandemic, an online purchasing pilot allowing consumers with low-incomes to use Supplemental Nutrition Assistance Program (SNAP) benefits for online grocery shopping in several states led to an 8% reduction in the prevalence of food insufficiency (4).

However, the online food environment also presents new challenges for supporting consumers in making informed, healthy choices. Online food shoppers lack consistent access to the same product information as in-store shoppers (5, 6) and face an onslaught of marketing messages, often aimed at luring them to purchase less healthy products (7). Food companies also advertise through social media (8) and other online settings like children’s educational websites (9), often targeting children who are particularly vulnerable to such marketing (10).

There is an urgent need for new policies to help ensure the internet is a force for good. Many U.S. food laws were written before the internet and are an uneasy fit for the digital age. While Congress can pass new legislation to improve the online food environment, existing laws give ample authority for federal regulators—like the U.S. Department of Agriculture (USDA), U.S. Food and Drug Administration (FDA), and Federal Trade Commission (FTC)—to adapt old laws to new environments. The Supreme Court recently struck down the *Chevron* judicial doctrine (11), denying any special deference to agencies in interpreting their own statutes, and expanded the “major questions” doctrine (12), preventing agencies from taking new actions of “vast economic and political significance” without clear direction from Congress. But federal regulators still have the ability to apply existing laws to emerging industries. State and local governments can also adopt policies to strengthen consumer protections when federal law does not preempt such policies.

This article presents legally feasible policy opportunities to promote food access, transparency and fairness, and nutrition in three settings: online grocery retail, online restaurant ordering, and marketing on social media and other online platforms.

## Online grocery retail

Online grocery shopping could improve food access, especially for the 40 million Americans living in areas with low income and low food access (13). The brick-and-mortar retail environment could still be improved to promote healthy foods, but healthy choices are even less clear in online grocery retail. Nutrition information can be hard to find for food sold online (5). Further, food and beverage manufacturers pay grocery stores large amounts of money to promote and place predominantly unhealthy products in prominent store locations (14), both in store (15) and online (16).

### Increasing food access

In 2023, 42.2 million Americans participated in SNAP (17) and another 6.6 million participated in the Special Supplemental Nutrition Program for Women, Infants, and Children (WIC) (18). These essential USDA programs support healthy food access for people with low incomes. The option of using SNAP and WIC benefits online further facilitates food access and offers program participants an equitable shopping experience. It’s a particularly important option for people who live in urban and rural food deserts (19), are immunocompromised, have mobility limitations, lack access to or funds for transportation, or are single parents.

Online SNAP purchasing is now available in all 50 states and Washington D.C. (20). In April 2023, 3.7 million households shopped online using SNAP. However, SNAP participants cite delivery and service fees as a barrier to utilizing their benefits online (21). USDA should consider reimbursing smaller, independent retailers for online delivery and service fees and requiring larger retailers to waive these fees for SNAP purchases.

Unlike online SNAP, online WIC purchasing is not yet widely available, but it would be especially beneficial because pregnancy and small children introduce challenges for in-person shopping. Current program rules technically prohibit using WIC benefits online (22), although the 2021 American Rescue Plan Act granted USDA authority to issue waivers relaxing these regulations, allowing funding for 7 states and one tribe to implement WIC online purchasing pilots (23). USDA should finalize its proposed rule from February 2023 that would modernize WIC to allow for online ordering in all states (24).

### Increasing information access

Currently, information required on food packaging to promote transparency and inform consumers’ choices is not always available to online shoppers. Even when available, it can be difficult to read, hard to find, incomplete, or inaccurate (5). Absent or inaccurate information poses a particular risk to people with food allergies and those managing diet-related chronic conditions.

In April 2023, FDA issued a Request for Information regarding food labeling in online grocery shopping (25). Consumer health advocates asked FDA to encourage or require online food retailers to disclose key product information at the online point of sale and to provide specifications for format and accessibility (26). Industry stakeholders shared challenges facing the information supply chain, such as the lack of uniform standards for when, how, and to whom suppliers must provide new or updated product information and images when there are new products or changes to existing products’ labels or formulations, and when and how online sellers must update the information on their webpages.

To improve transparency and information access for online grocery shoppers, FDA and USDA should issue guidance encouraging online sellers to ensure that the same nutrition, ingredient, and allergen information available in stores is available, easy to find, and easy to read at the online point of sale. To address challenges facing the food industry in managing the transfer of food information, the guidance should establish best practices for how and when product information and images should be transmitted from manufacturer to retailer to consumer. It should also encourage product webpages to be configured with Americans with Disabilities Act (ADA) compliance and accessibility in mind. Finally, it should encourage sellers of recently reformulated products with multiple formulations in commerce at the same time to alert consumers to this fact and provide consumers with product information for every version they might receive.

New federal legislation could mandate compliance with such guidance by all food sellers (27). However, to make online labeling mandatory for many retailers without new legislation, USDA could issue regulations making available, accessible online information a requirement for SNAP- and WIC-authorized online retailers. This would affect the majority of retailers, as about 257,000 U.S. food retailers (28) (84% of all U.S. food retailers) (29) accept SNAP. All online shoppers (those using SNAP benefits and other forms of payment) would have increased access to information. To support retailers, USDA should assist with data management and webpage updates through the SNAP EBT Modernization Technical Assistance Center (30).

### Encouraging healthy choices

There are numerous policies underway aimed at supporting consumers in making healthier choices at the grocery store. These policies must consider both in-store and online settings. One example is FDA’s proposed regulation establishing a mandatory, standardized front-of-package nutrition labeling (FOPNL) system to help consumers, particularly those with lower nutrition knowledge, quickly and easily identify foods that can help them build a healthy eating pattern (31). This policy initiative is described in detail elsewhere (31, 32). FDA’s regulations, or subsequent guidance, should specify that FOPNL should appear on all images of the product’s principal display panel displayed on product webpages.

Another example is state and local policies requiring healthy placement standards for food retail establishments to make it easier for customers and their children to avoid marketing and impulse purchases of drinks and snacks high in sugar and salt. Berkeley (33) and Perris (34), California have passed policies requiring all large retailers to place only healthy foods and beverages in checkout aisles. Similar future policies should specify how these requirements apply online and should also be considered at the federal level. To benefit consumers in all 50 states without necessitating federal legislation, USDA should consider establishing mandatory healthy food marketing and product placement standards for SNAP-authorized retailers (21). FTC should consider issuing guidance for retailers on how to place foods and beverages in the checkout aisle—including the virtual equivalent of the checkout aisle. The Farm Bill should authorize pilot projects to test and inform the implementation of such requirements for SNAP-authorized retailers.

## Online restaurant ordering

Restaurant foods in the U.S. are often high in calories, sodium, and added sugars (35). FDA requires calorie disclosures on all menus from U.S. chain restaurants with 20 or more locations, Philadelphia requires sodium warnings on chain restaurant menu items that contain more than a day’s worth of sodium (2,300 milligrams), and New York City (NYC) requires those same sodium warnings plus warnings on chain restaurant menu items containing more than a day’s worth of added sugars (50 grams) (36–39). These labeling policies can improve the nutritional quality of food orders (40, 41) and encourage restaurants to sell healthier items (42). To have their full intended benefit, requirements for nutrition information and safety warnings must apply to menus regardless of where people view them, whether in a physical restaurant, on a website, or on a third-party ordering app.

### Federal calorie labeling

FDA regulations explicitly apply menu labeling requirements to “menus on the Internet” (43). These regulations clearly cover menus on restaurants’ websites and mobile apps, but whether those same menus are still covered when they are posted or maintained by restaurants on third-party platforms (TPPs) has been a topic of debate. In 2023, FDA published a draft guidance stating that calorie labeling for menus on TPPs is voluntary, implying that such menus are exempt from federal menu labeling requirements (44). A coalition of consumer health organizations pushed back, filing a petition calling on FDA to make calorie labeling for menus on TPPs mandatory, not voluntary, in their final guidance and arguing that the agency has full authority to do so (45).

The argument over FDA authority amounts to a question of control. While TPPs themselves are not subject to FDA’s regulatory authority, FDA does have authority to regulate menus that are posted by restaurants covered under the federal menu labeling law. Therefore, advocates contend that FDA can require restaurants that post and control menus on TPPs to include calorie information. All major TPPs allow restaurants to upload and update their own menus on the platforms through merchant portals (45), plus Grubhub and DoorDash have stated that the majority of orders on their platforms come from partnered restaurants (i.e., restaurants that have agreed to partner with the TPP by posting and updating their menus using the merchant portal) (46). Thus, covered restaurants should be required to comply with calorie labeling when they post menus on a TPP, just as they must comply when they post menus on a proprietary website or mobile app.

Applying federal menu labeling requirements to menus on TPPs is essential for ensuring consistent calorie information access. While there is strong compliance with calorie disclosure requirements on printed menus and website menus (47), the prevalence of calorie labeling on TPPs is currently low (6). A 2022 study found at least one calorie disclosure on only 58% of menus on DoorDash, 51% of menus on Uber Eats, and 12% of menus on Grubhub (6).

To increase compliance with federal menu labeling requirements, FDA should issue final guidance clarifying that the requirements do apply to menus that covered restaurants post on TPPs.

### State and local nutrient warnings

Similar to FDA’s calorie labeling regulations, local nutrient warning policies apply to menus on restaurant websites and mobile apps, but so far have not been interpreted to apply when those same menus are posted on TPPs. Philadelphia’s guidance to restaurants says sodium warning labels must appear on websites and mobile applications if the covered establishment takes orders using these methods, but they are “not required on third-party sites (e.g., Uber Eats, Caviar, Grubhub)” (48). NYC’s policy exempts “third party online ordering sites, such as Seamless or Grub Hub, because the companies operating these websites are not food service establishments with a Health Department permit” (37).

As additional policies are developed to warn consumers about menu items with extremely high levels of sodium and other nutrients like added sugars, they should include menus on TPPs. Furthermore, NYC and Philadelphia policymakers should encourage TPPs to provide the capability to add warnings beside menu items through their merchant portals and should encourage merchants to utilize this feature.

## Marketing on social media and other online platforms

Children’s exposure to marketing via social media and other online platforms is concerning because food marketing exposure can impact children’s food preferences, choices, and intake (49), and their ability to understand the persuasive intent of advertising is not yet fully developed (10). Much of the food marketed to kids online is unhealthy. In one study, 72% of children and adolescents were exposed to food and beverage advertising on social media apps, and the most common exposures were to ads for fast-food restaurants, sugar-sweetened beverages, and candy and chocolate (50).

The FTC Act allows FTC to regulate unfair and deceptive business practices, including certain online marketing activities (51). FTC’s Endorsement Guides address unfair and deceptive advertising practices in advertisements containing endorsements, defined as “any advertising, marketing, or promotional message for a product that consumers are likely to believe reflects the opinions, beliefs, findings, or experiences of a party other than the sponsoring advertiser” (52), including—for example— social media posts from paid influencers. In 2023, FTC updated the Endorsement Guides to include a section titled “Endorsements Directed to Children,” which notes that practices that may be appropriate in content aimed at adult audiences may not be appropriate in content aimed at children (53). However, FTC does not provide practical advice for handling endorsements in content specifically aimed at children, stating only “Practices that would not ordinarily be questioned in advertisements addressed to adults might be questioned in such cases” (53). The agency should better protect children from unfair unhealthy food and beverage advertising online by providing more specific language on best practices for endorsement disclosure for advertisements aimed at children.

The FTC Act’s “6(b) authority” also empowers FTC to require businesses to file reports and answer questions about their business practices, and to publish wide-ranging agency studies based on such reports and information (54–56). FTC used its 6(b) authority to conduct investigations and publish reports on food industry expenditures on marketing to children in 2008 and 2012 (57, 58). These reports provided insight into companies’ spending on marketing to children, which products are most frequently advertised, and through which channels children are exposed to food advertising (57, 58). However, the most recent of these reports used data from 2009 (58). FTC should publish an updated food-marketing-to-kids report, paying special attention to digital marketing, including how companies may be disproportionately advertising to specific demographic groups and marketing on online learning platforms. This report would help inform regulatory, legislative, and industry self-regulatory activities.

Finally, state governments should leverage their consumer protection statutes and pass policies to strengthen them. For example, New York’s proposed Predatory Marketing Prevention Act would direct courts to consider the age of the target audience and other factors that might affect comprehension in determining when food marketing (which includes online marketing) is false or misleading, enhancing the state attorney general’s ability to prosecute companies for such predatory marketing (59).

Nutrition misinformation is another serious concern in the online food marketing environment. FDA and FTC have limited authority to address false and misleading statements that are not tied to the promotion of specific products or brands, but a clear step FTC can take to promote public trust in advertising is to take action more often against social media influencers who fail to disclose sponsored content. While such actions cannot eliminate misleading information from the internet, transparency regarding funding for endorsements helps consumers evaluate the trustworthiness of the message. In 2023, the agency sent warning letters to the American Beverage Association, Canadian Sugar Institute, and 12 nutrition influencers for failing to employ required disclosures on paid advertisements that promoted the safety of aspartame and sugary products (60). FTC should continue to monitor and take action against such violations and establish a means for the public to easily report known or suspected violations.

## Discussion

As the online food environment becomes increasingly influential in guiding consumers’ access to food and information, federal regulators and state and local lawmakers should take advantage of the many policy opportunities to promote food access, transparency, and healthy choices through online food sales and marketing.

In doing so, policymakers must consider the challenges and potential unintended consequences of implementing new policies (see Table 1).

**Table 1:** Recent actions and policy recommendations to promote food access, transparency, and healthy food choices online.

Domain	Level of government or agency	Recent actions (past 10 years)	Recommendation	Challenges
Online grocery retail	FDA	Hosted a summit on Ensuring the Safety of Foods Ordered Online and Delivered Directly to Consumers (October 2021) (61) Issued a Request for Information about food labeling in online grocery shopping (April 2023) (25)	Issue guidance encouraging online sellers to ensure that the same nutrition, ingredient, and allergen information that would be available in stores is available, easy to find, and easy to read at the online point of sale. Specify in regulations or guidance that front-of-package nutrition labeling should appear on all images of products’ principal display panels displayed on product webpages from online sellers.	FDA lacks clear authority to mandate information at the online point of sale for all foods. Voluntary guidance may not ensure high compliance and would not be enforceable. FDA has limited resources to enforce food labeling requirements. Front-of-package nutrition labeling faces substantial opposition from food industry stakeholders.
	FTC	Launched an inquiry (November 2021) (62) and released a report (March 2024) (63) on grocery supply chain disruptions, including anticompetitive retailer marketing practices	Issue guidance on retailers not placing unhealthy foods and beverages in the checkout aisle—including the virtual equivalent of the checkout aisle.	FTC has limited resources to address all consumer protection concerns. Online placement regulation faces substantial opposition from food industry stakeholders. Requires coordination between retailers and manufacturers to identify products for promotion that meet nutrition criteria.
	USDA	Funded SNAP online purchasing pilots (2019–2023) (64) Announced that SNAP online purchasing is available in all 50 states (June 2023) (20) Proposed regulations to modernize WIC and allow online ordering in all states (February 2023) (24)	Reimburse small, independent retailers for online delivery and service fees for SNAP purchases, and require larger retailers to waive these fees. Issue final regulations to allow online ordering using WIC benefits. Require SNAP- and WIC-authorized retailers to: Ensure that the same nutrition, ingredient, and allergen information that would be available in stores is available, easy to find, and easy to read at the online point of sale. Adhere to healthy food product placement standards both in-store and in the online setting.	USDA has limited resources and authority to reimburse retailers for online delivery and service fees. Large retailers may oppose requirements to waive fees for SNAP purchases, and could avoid covering these fees by declining to accept SNAP as a form of online payment. The SNAP EBT Modernization Technical Assistance Center (30) would need additional funding to support retailers with implementing online labeling requirements. Retailers that face challenges complying with online labeling requirements could avoid facing these requirements by declining to accept SNAP as a form of online payment. Retailers would need to work with manufacturers to ensure timely sharing of complete and accurate label information and to identify products for promotion that meet nutrition criteria.
	State/local government	Berkeley, CA (2020) (33) and Perris, CA (2023) (34) passed ordinances requiring healthy checkout aisles (in-store only)	Ensure that policies establishing healthy food product placement standards specify how these requirements apply in the online setting.	State/local governments may face pushback when trying to regulate online settings that are used both within and outside of their jurisdictions.
Online restaurant ordering	FDA	Issued draft guidance encouraging calorie labeling for menus on third-party ordering platforms (December 2023) (44)	Issue final guidance clarifying that federal menu labeling requirements apply to menus that covered restaurants post on third-party ordering platforms.	It is not always clear whether a covered restaurant or a non-covered third party controls the content of an online menu, and FDA can only require calorie labeling for menus controlled by covered restaurants.
	State/local government	New York, NY (2015) (37) and Philadelphia, PA (2018) (36) passed policies requiring sodium warnings on chain restaurant menus New York, NY passed policies requiring added sugars warnings on chain restaurant menus (2022–2023) (38, 39) State lawmakers in NY, NJ, and MA introduced sodium and/or added sugar warning policies (2015–2024) (65–67)	Include all online menus, including menus on third-party ordering platforms, in policies requiring sodium and added sugar warnings.	To date, no policies requiring sodium or added sugars warnings on restaurant menus have included menus on third-party platforms.
Marketing on social media and other online platforms	FTC	Updated the Guides Concerning the Use of Endorsements and Testimonials in Advertising (July 2023) (53) Sent warning letters to companies and nutrition influencers that failed to employ required disclosures on paid advertisements (November 2023) (60)	Issue guidance on what practices should not be used in advertisements aimed at children, including web advertisements. Publish an updated report on food marketing to kids, with special attention to digital marketing, disproportionate advertising to specific demographic groups, and use of online learning platforms. Take action against social media influencers who fail to disclose sponsored content and establish a means for the public to easily report known or suspected violations.	Strong opposition from the advertising industry may limit FTC’s willingness to craft rules addressing advertising to children. More research is needed to understand the impact of disclosure on advertising’s influence in children. It can be difficult for FTC to monitor the disclosure practices of the many social media influencers paid to promote products.
	State government	Lawmakers in NY introduced the Predatory Marketing Prevention Act* (March 2023) (59)	Pass policies to strengthen consumer protection policies by deeming unhealthy food marketing (including online marketing) directed at vulnerable groups, like children and older adults, to be false and misleading.	Opposition from the food, beverage, and advertising industries may dissuade state legislatures from acting. State consumer protection laws vary leading to varying abilities across states to enact policies addressing false and misleading food marketing.

*Policy introduced but not passed into law as of July 2024. FDA, Food and Drug Administration; USDA, U.S. Department of Agriculture; FTC, Federal Trade Commission; SNAP, Supplemental Nutrition Assistance Program; WIC, Special Supplemental Nutrition Program for Women, Infants, and Children.

FDA and FTC have limited resources for policy enforcement. State and local governments may face pushback when adopting policies that apply to online settings accessed from both within and outside of their jurisdictions. Retailers could decide not to accept online SNAP payments to avoid complying with labeling and marketing requirements tied to online SNAP, thus negating recent improvements in food access from online SNAP expansion. New food labeling and marketing requirements often face opposition from the food industry, including threats to increase food prices. And policies restricting food marketing to kids online must contend with privacy concerns, such as balancing the need to know the age of users with the risks of collecting personal information. Fortunately, these challenges are not insurmountable and policies can be creatively and flexibly designed to avoid such unintended consequences.

Future research should explore policies outside the scope of this article but related to consumer protection in the online food environment, including those seeking to decrease prices of food sold online (e.g., commission fee caps and price discrimination), improve wages and workplace protections for food delivery workers, increase food safety (e.g., safe handling protocols for delivery drivers and bikers), and ensure data privacy and security (e.g., protection of user data and prohibition of unfair ad targeting based on factors such as SNAP status or age).

The online food environment presents myriad challenges and opportunities for improving food access, transparency, and nutrition. Federal, state, and local policymakers must meet the moment and ensure adequate regulation of online spaces to support consumers in making informed, healthy choices.
